# Improving outcomes for hospital patients with critical bleeding requiring massive transfusion: the Australian and New Zealand Massive Transfusion Registry study methodology

**DOI:** 10.1186/s13104-016-2261-6

**Published:** 2016-10-06

**Authors:** J. C. Oldroyd, K. M. Venardos, N. J. Aoki, A. J. Zatta, Z. K. McQuilten, L. E. Phillips, N. Andrianopoulos, D. J. Cooper, P. A. Cameron, J. P. Isbister, E. M. Wood

**Affiliations:** 1 Transfusion Research Unit, Department of Epidemiology and Preventive Medicine, Monash University, Clayton, VIC 3004 Australia; 2 Centre of Research Excellence for Patient Blood Management in Critical Illness and Trauma, Monash University, Clayton, VIC 3004 Australia; 3 Emergency and Trauma Centre, The Alfred Hospital, Melbourne, VIC 3004 Australia; 4 Department of Haematology, University of Sydney, Royal North Shore Hospital, St Leonard, Sydney, NSW 2065 Australia; 5 Department of Epidemiology and Preventive Medicine, Monash University, Clayton, VIC 3004 Australia

**Keywords:** Critical bleeding, Massive transfusion, Registry

## Abstract

**Background:**

The Australian and New Zealand (ANZ) Massive Transfusion (MT) Registry (MTR) has been established to improve the quality of care of patients with critical bleeding (CB) requiring MT (≥ 5 units red blood cells (RBC) over 4 h). The MTR is providing data to: (1) improve the evidence base for transfusion practice by systematically collecting data on transfusion practice and clinical outcomes; (2) monitor variations in practice and provide an opportunity for benchmarking, and feedback on practice/blood product use; (3) inform blood supply planning, inventory management and development of future clinical trials; and (4) measure and enhance translation of evidence into policy and patient blood management guidelines. The MTR commenced in 2011. At each participating site, all eligible patients aged ≥18 years with CB from any clinical context receiving MT are included using a waived consent model. Patient information and clinical coding, transfusion history, and laboratory test results are extracted for each patient’s hospital admission at the episode level.

**Results:**

Thirty-two hospitals have enrolled and 3566 MT patients have been identified across Australia and New Zealand between 2011 and 2015. The majority of CB contexts are surgical, followed by trauma and gastrointestinal haemorrhage. Validation studies have verified that the definition of MT used in the registry correctly identifies 94 % of CB events, and that the median time of transfusion for the majority of fresh products is the ‘product event issue time’ from the hospital blood bank plus 20 min. Data linkage between the MTR and mortality databases in Australia and New Zealand will allow comparisons of risk-adjusted mortality estimates across different bleeding contexts, and between countries. Data extracts will be examined to determine if there are differences in patient outcomes according to transfusion practice. The ratios of blood components (e.g. FFP:RBC) used in different types of critical bleeding will also be investigated.

**Conclusions:**

The MTR is generating data with the potential to have an impact on management and policy decision-making in CB and MT and provide benchmarking and monitoring tools for immediate application.

## Background

Blood transfusion is a common hospital procedure and may be life-saving. However, it is not without risk. Known risks include transfusion-associated infectious diseases, haemolytic transfusion reactions, allergic reactions and transfusion-related acute lung injury [1]. Other adverse outcomes associated with transfusion, albeit from observational studies, include longer duration of intensive care unit (ICU) and hospital admission [2], multi-organ failure [3] and higher mortality [4].

Advances in critical care and surgical techniques have resulted in more patients with critical bleeding (CB) who require large volume blood transfusion support [5]. These “massive transfusions” (MT) have variously been defined as 10 or more units of red blood cells (RBC) transfused in 24 h or the “transfusion of half of one blood volume in 4 h, or more than one blood volume in 24 h” (adult blood volume is approximately 70 ml/kg) [6]. MT are important because the risks of transfusion are amplified when larger volumes of products are administered [7–9]. The consequences of variations in MT practice on patient outcomes are unknown. Reported mortality rates for patients requiring MT are between 25 and 48 % [10–14]. Massive transfusion also poses logistical challenges for blood services/laboratories/hospitals as CB requiring MT is often unpredictable. These challenges relate to the need to have blood available—including Group O RhD negative RBCs and other blood components such as fresh frozen plasma (FFP), cryoprecipitate and platelets.

The evidence base for transfusion practice is incomplete, particularly in patients with CB. In 2011, Australia’s National Blood Authority (NBA) published patient blood management (PBM) Guidelines for CB [6] which summarised the available evidence and made recommendations for practice where the body of evidence was sufficient. Where there was insufficient evidence, “practice points” were developed though a consensus-based process to guide clinical practice. The PBM Guidelines identified major evidence gaps including: (1) the role of RBC transfusion; (2) dose, timing and ratio of component therapies; (3) effect of non-transfusion interventions; and (4) impact of blood component therapies on patient outcomes as particularly important [6]. Although high quality randomised controlled trials have recently been published [15–17], practices such as the routine administration of a 1:1 ratio of RBC to FFP [10, 11, 18–21] and greater use of recombinant activated factor VII (rFVIIa) are originally derived from the trauma setting without randomised controlled trial evidence. They have subsequently been extrapolated to non-trauma settings despite important differences in the pathophysiology of CB events in other settings, especially obstetrics [22]. Currently, there is no process for the systematic evaluation of compliance with the PBM Guidelines. In addition, there are major challenges associated with supplying blood products across Australia and New Zealand given their geographies, and a dearth of information available on clinical outcomes associated with blood transfusion.

Clinical quality registries are one of the most effective means of monitoring and encouraging uptake of healthcare guidelines [23]. They lead to improved quality of care by providing clinicians with credible risk-adjusted outcome data, enabling them to benchmark their outcomes against local and international data [24–26]. Given the incomplete evidence base in CB, the need for data on practice/blood product use in Australia and New Zealand and the current lack of monitoring of variations in practice, we established the Massive Transfusion Registry (MTR) to improve the quality of care of patients with CB requiring MT.

## Methods

### Study aims

The MTR was established to: (1) improve the evidence base for transfusion practice by systematically collecting data on MT practice and clinical outcomes; (2) monitor variations in practice and provide an opportunity for benchmarking, feedback on practice/blood product use, quality and safety in hospital practice and accreditation; (3) inform blood supply planning, inventory management and development of future clinical trials; and (4) measure and enhance translation of evidence into policy and PBM Guidelines.

### Health care systems in Australia and New Zealand

Health care systems in Australia and New Zealand consist of public and private providers, including hospitals, primary health care, clinicians, nurses, other health professionals, and government and non-government organisations [27, 28]. They deliver many services for the prevention and treatment of diseases. In both countries, government funds public sector health services and private health service providers are owned and operated by the private sector.

### Overview of Australia and New Zealand blood bank networks

The blood bank networks in Australia and New Zealand consist of several interconnected organisations involved in the supply and management of blood and blood products. National blood services providing allogeneic components and fractionated plasma products are operated by the Australian red cross blood service or New Zealand blood service, funded by national governments. There are national regulators (e.g. Therapeutic Goods Administration in Australia and Medsafe in New Zealand) and funding is coordinated at a national level (e.g. National Blood Authority in Australia). Specialist societies (e.g. ANZ society of blood transfusion) and specialist colleges (e.g. Royal Australasian College of Physicians) provide education and training in clinical and laboratory transfusion practice. Commonwealth serum laboratories (CSL) Behring Australia provides plasma fractionation services to both countries. Hospital and private pathology services operate hospital blood banks for storage, crossmatching and issue of blood.

### Governance

A steering committee oversees the conduct, development and outputs from the registry. The steering committee includes practising clinicians (haematologists, intensivists, emergency physicians, obstetricians, anaesthetists), a statistician and representatives from blood sector partner organisations. Terms of reference for the MTR steering committee, a data access and publications policy and a communications plan are in place. Access to aggregate data is provided to steering committee members. Each party agrees to treat the data in accordance with their obligations under their applicable national legislation for intellectual property and privacy. External interested parties, including local investigators at participating sites and government agencies, submit formal data requests to the steering committee for approval prior to being granted data access.

### Ethics, consent and permissions

Ethical approval to establish the MTR was granted by Monash University Human Research Ethics Committee. In addition, ethical approval to collect identifiable patient level data has been obtained from all 32 participating hospital sites. It was not practical to obtain individual patient consent to participate because the cases are unpredictable, many are emergencies, and there is a high early mortality in CB/MT. Therefore, a waived consent model was chosen. The MTR qualifies for the conditions for waived consent as outlined in the NHMRC National Statement on Ethical Conduct in Human Research on the basis that: (1) involvement in the registry carries no more than low risk to participants (2) the benefits from the registry justify any risks of harm associated with not seeking consent (3) it is impracticable to obtain consent (4) there is no known or likely reason for thinking that participants would not have consented if they had been asked, and (5) there is sufficient protection of their privacy and an adequate plan to protect the confidentiality of data. Consent waiver was also suitable on the basis that: (1) Australian Commission on Safety and Quality in Health Care guidelines suggest that complete data must be collected from the entire eligible population in order to minimise selection bias [29], (2) obtaining written consent is impractical for many registries [30], and (3) consent wavier improves case-capture [26].

The MTR collects unique patient identifiers (medical record number, full name, date of birth and gender) which are used for the sole purpose of data linkage.

### Study population

Patients (≥18 years) at participating hospitals are included if they receive ≥5 units RBC within any 4-h period of hospital admission [31]. This definition was chosen after verification that it optimised case-capture [31]. This was necessary because no standard definition of MT exists in the international literature and use of some definitions may lead to bias. For example, defining MT as 10 or more units of RBCs in 24 h (10/24 h) may exclude trauma patients who die within the first 24 h (‘survivorship bias’) whereas patients who do not require blood early on, but have a high cumulative transfusion requirement over a longer period, may be disproportionately represented (‘catch-up’ bias). These may be overcome by the use of time-dependent MT definitions (e.g. ≥5 units RBC in 4 h and ≥6 units RBC in 6 h), which focus on acuity of MT requirements during the resuscitation period (in the first 2–6 h following injury). We performed a validation study to examine the completeness of capture of CB events using three different definitions of MT (5U RBC in 4 h; 6U RBC in 6 h; 10U RBC in 24 h) [31]. The most inclusive definition with minimal bias was the 5U RBC in 4 h, which captured 94 % of all CB events and all types of CB events, including obstetric haemorrhage. The least inclusive definition was the 10U RBC in 24 h with less than 50 % of patients identified. Consequently, the registry uses 5U RBC in 4 h to define MT.

Given the difficulty of measuring bleeding reliably and identifying when CB occurs across many different clinical contexts and hospital sites, eligible patients are identified using a computer-generated algorithm to query hospital blood bank databases to ensure a centralised and systematic approach. Each hospital created its own queries based on the information system specific to the hospital. Hospital participation was contingent on hospitals having the data informatics capability to run these scripts and manage large volumes of transfusion data. Common informatics problems encountered were the inability to develop or run a script to identify MT patients, inability to conform to the data extraction template, and informatics systems which required manual extraction of laboratory data.

### Data items

MTR data items are extracted for each patient for each hospital admission. A hospital admission represents a patient’s entire hospital stay and is demarcated by an admission date (the date they were admitted to hospital) and a separation date (the date they were discharged from hospital or the date of their death). Some patients will also have episodes of care (EOC), which are phases of treatment received by a patient within their hospital admission. An EOC ends when the principal clinical intent changes or when a patient formally separates from the hospital. There can be more than one EOC within one admission.

**Fig. 1:**
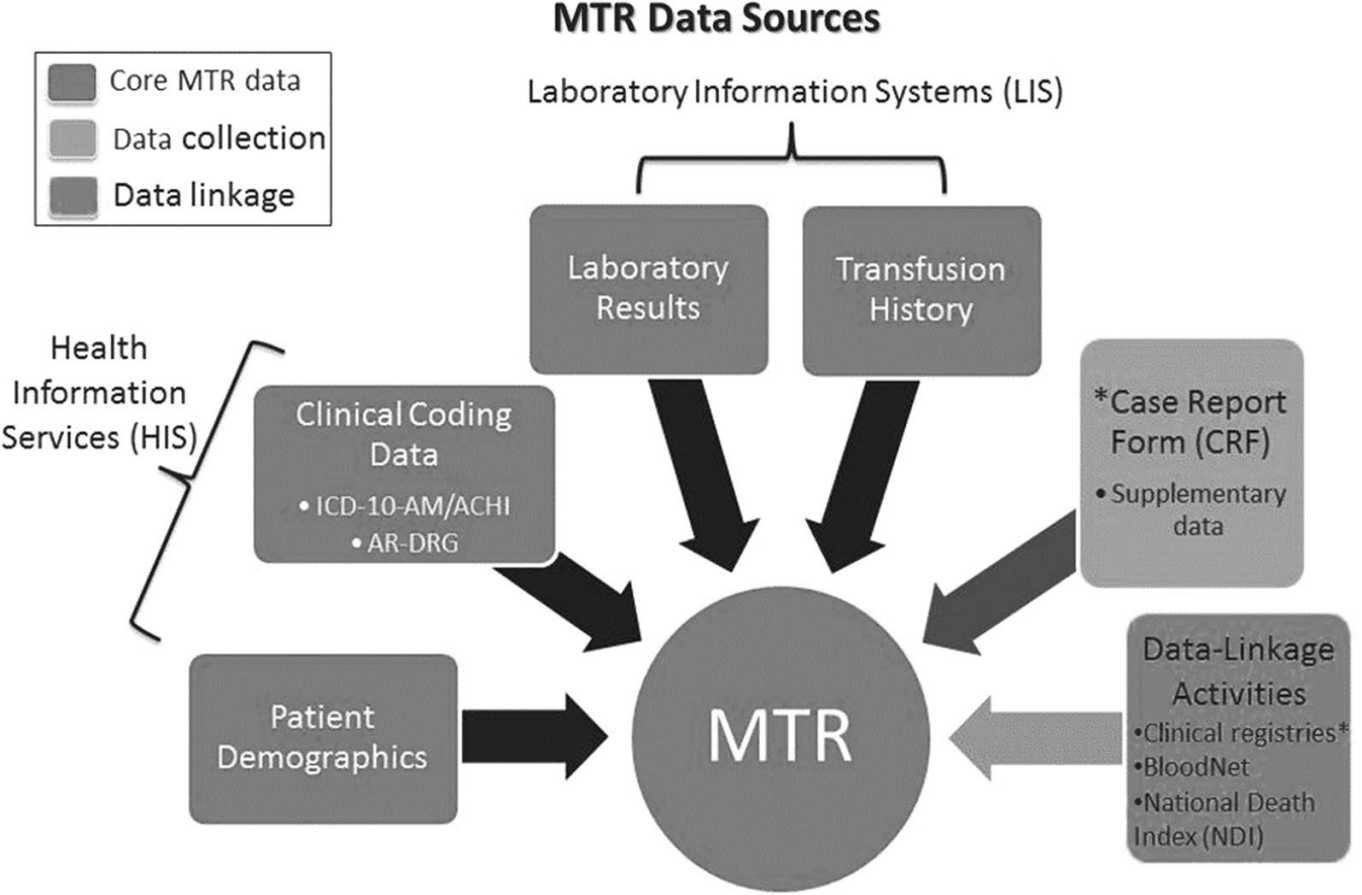
MTR data sources

The MTR uses existing, electronically stored clinical data from hospital information systems. MTR data are captured within three packages (see Fig. 1, Table 1): (1) Patient demographic and outcome data in conjunction with diagnosis and procedure clinical coding from the hospital information services (HIS) or patient administration system; (2) full transfusion history for a patient’s hospital admission including information on all fresh blood products, fractionated plasma products, and adjunctive therapies. Both Australia and NZ have nationally standardised products for processes like leucodepletion (100 %). Red cells are all whole blood-derived in both countries. Platelets are both whole blood or apheresis-derived, but they are a standardised product in terms of manufacturing specifications. Only products transfused (not issued then returned unused), are stored within the registry; and (3) Laboratory results for the patient’s hospital admission, both pre- and post-MT, from Laboratory Information Systems (LIS). Unique patient identifiers, required for verification and linkage, are collected in each package.

**Table 1:** MTR data items

Health information services	Transfusion history	Laboratory test results
Facility identifier	Facility identifier	
Medical record number	Medical reference number	*p*O2
Surname, first name	Name	*p*CO2
Date of birth	Date of birth	pH
Gender	Gender	H Ion concentration
Admission number	Product lot number	Calculated bicarbonate
Episode of care number	Type of product: RBC, platelets, fresh frozen plasma, cryoprecipitate, Prothrombinex-VF, fibrinogen concentrate, rFVIIa	Sodium potassium chloride
Episode sequence number	Donation number	Anion gap
Date of hospital admission	Expiry date of product	Measured ionised calcium
Time hospital admission	Unit ABO	Base deficit (or excess)
Date of episode of care admission	Only products issued (and not returned) or transfused	
Time of episode of care admission	Number of units or volume issued	INR
Completion date of episode of care	Place issued i.e. ward, theatre, ICU	Prothrombin time
Completion time of episode of care	Date issued	APTT
Date of death or discharge	Time issued	Fibrinogen level
Time of death or discharge	The age of the blood issued	D-Dimer (if available)
Name of transferring hospital		
Admission type		Haemoglobin
Discharge unit		White blood cell count
Patient status at discharge		Platelet count
Transfer destination		Red blood cell count
Hospital length of stay (hrs)		Haematocrit
ICU length of stay (hrs)		MCV
Ventilation time		MCHC
Primary clinical specialty		
Sequence of diagnoses codes		Sodium
ALL diagnoses ICD-10-AM codes		Potassium
Descriptions of diagnoses codes		Chloride
Qualifier for each ICD-10-AM code		Bicarbonate
Condition onset flag		Urea
ALL procedure ACHI codes		creatinine
Descriptions of procedure codes		
Procedure dates		Bilirubin
Sequence of procedure codes		ALP
DRG type		ALT
		Gamma GT
		Albumin
		Protein Total
		Positive or negative for bacterial infection
		Lactate
		Estimated glomerular filtration rate
		Blood group and antibody screen

*ICU* intensive care unit, *ICD10* international classification of disease 10, *ACHI* Australian classification of health interventions, *DRG* diagnosis-related group, *RBC* red blood cells, *rFVIIa* recombinant activated factor VIIa, *INR* international normalised ratio, *APTT* activated partial thromboplastin time, *MCV* mean cell volume, *MCHC* mean cell haemoglobin content, *ALP* alkaline phosphatase, *ALT* alanine phosphatase, *Gamma GT* gamma-glutamyl transpeptidase

#### Derived variables

Derived variables are generated within the registry using raw data. They are generated automatically for speed, accuracy and efficiency. They include the Charlson Comorbidity Index (CCI) to estimate disease burden [32, 33]; counts of ICD10 diagnosis codes; unique bleeding contexts within an EOC; counts of each transfusion product, laboratory tests for each EOC and survival status on discharge and 24 h post-MT.

### Data management

**Fig. 2:**
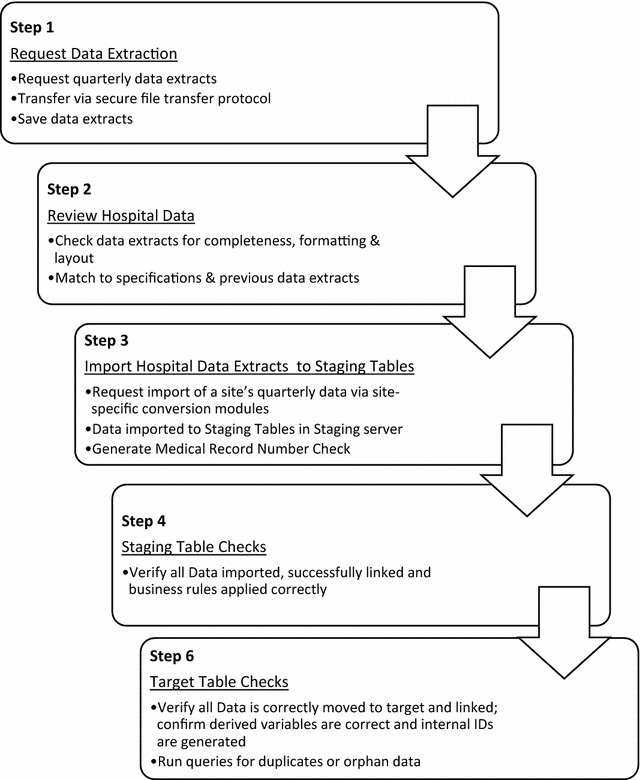
Flowchart of data extraction into the MTR

Requests for data extraction are made retrospectively on a quarterly basis from data custodians at participating sites. Retrospective recruitment ensures availability of all data items at the time of extraction, especially clinical coding data. All data extracts from sites are transferred via password protected secure file transfer protocol. Subsequent data processing involves source file verification for file completeness, formatting and layout (Fig. 2). Site-specific conversion modules have been created and are used to import the data packages. The conversion modules mean that hospitals need to extract data in the same way each quarter. Data are imported into the database into ‘staging’ and ‘target’ tables which are accessible via remote server. These table views provide opportunities to check for discrepancies and inconsistencies within hospital datasets and whether data from all three packages (HIS, transfusion history and LIS) have been successfully linked. Staging table checks include checks to ensure that specific rules to clean data have been applied; that there has been correct linkage; that mapping of various codes from reference or look-up tables built within the database has occurred; and that consistent terminology and descriptions of variables for all sites have been assigned. Target table checks include the application of unique constraints to remove any duplicates and generate a number of derived variables using the raw cleaned data contained within the various tables. Target checks also show whether the database has assigned unique internal patient identification numbers associated with unique episode IDs, which are in turn associated with unique HIS, transfusion history and LIS results. Verification queries in the target server are also run to check for orphan data. Following these quality assurance checks the data is deployed to the ‘production’ table from which data cuts are taken for all reporting and analyses.

### Quality assurance

Quality assurance measures are applied to data item selection, requests for data items, data coding and data entry (Fig. 2). Data items selected are standardised across sites and are provided in most cases by personnel trained in data management such as business analysts, HIS staff or information technology managers. Requests for data items are standardised in project protocols. The data importing process includes extensive source file checks, the application of business rules and verification of successful linkage. As data move through the registry to the production database, further checks are applied to verify that data are moving correctly, linked successfully, derived variables are correct and internal identification numbers are generated.

### Reporting

Hospital Data Reports to participating sites are generated bi-annually. They allow benchmarking of practice with comparable health services. Here, de-identified individual site data are presented and compared with national and overall MTR data. Data reports are also customised for individual sites by clinical bleeding contexts to facilitate meaningful comparison and to identify local targets for audit or clinical investigation.

External communication consists of a MTR Newsletter (for progress reports/updates, upcoming events and publications of interest), the Transfusion Outcomes Research Collaborative (TORC)/MTR website (for project details), a TORC Biennial Report (overview of study achievements), Annual Investigator meetings (strategic planning and networking), MTR special interest group meetings (for the investigation of specific clinical questions) and data reports to partner organisations (project updates to funders).

### Pilot data

The MTR builds on the Haemostasis Registry (2005–2010) which collected data on the off-label use of rFVIIa in CB from 96 hospitals and included approximately 3500 patients in Australia and New Zealand [34]. A pilot MTR study was undertaken to test the feasibility of collecting MT data at six hospitals and verified that much of the information required to analyse CB/MT events is already collected for other purposes. The pilot MTR developed mechanisms to identify MT patients at sites, extract relevant data from each source, securely transfer data to Monash University, link data from each source, and implement a coding framework to co-ordinate the large volume of data supplied. Validation studies were undertaken to test assumptions and methodologies and verify the data [31, 35].

### Research arising from the MTR

#### Validation study

A study limitation is that MTR transfusion time is the product issue time from the hospital blood bank, which will not always be the same as the exact time the patient was administered the product. Therefore, a validation study was conducted to examine whether hospital ‘time of issue’ of blood products from the blood bank is a reliable estimate of the ‘time of transfusion’ [35]. Good concordance was found with the median transfusion time for the majority of fresh products being ‘product event issue time’ from the blood bank plus 20 min (>30 min for FFP), which reflected the expected time to transport, check and prepare the transfusion. For the purpose of a registry, these results support the use of hospital blood bank computer records as an appropriate source of blood product information for linking with other data sources.

#### Data linkages

Data linkage between the MTR and the National Death Index (NDI) in Australia and the New Zealand Ministry of Health mortality data is performed annually to assess 30- and 90-day mortality. Collaborative linkage projects with other clinical registries available through the Department of Epidemiology and Preventive Medicine, Monash University are being explored to provide context specific information and additional outcomes. Data linkage with other clinical registries will allow case–control comparisons of in hospital mortality, hospital and ICU LOS and ventilation times with matched patients who did not receive MT.

Another opportunity that exists is linkage with Blood Net, Australia’s national online inventory management system. This would allow an exploration of questions relating to blood utilisation at the time of MT (including the number of group O, RhD negative RBC). Such a linkage would also allow an examination of blood utilisation for MT as a proportion of overall inventory according to institution type, geographical location and type of CB/MT events, and against PBM guidelines.

#### Supplementary data collection

Specifically designed sub-studies requiring supplementary data collection are underway. Transfusion nurses or transfusion quality/safety officers or other trained staff at participating sites collect the supplementary data.

#### Economic analyses

Health economic data will be collected in order to estimate costs to the health system of CB/MT, as well as modelling economic impacts of variation in practice and outcome, and estimates of cost savings and patient benefits that could be realised by improved practice. This will enable the conduct of cost-effectiveness analyses to determine values of different practices and/or therapies, such as the availability of pre-thawed FFP, use of tranexamic acid (TXA, an antifibrinolytic agent) and variations in inventory holdings.

### Future directions

Further recruitment of regional and rural sites will allow comparisons of clinical practices in non-urban settings where blood supply and administration practices may differ from metropolitan settings. A preliminary comparison suggests there are differences in Australian MT patients by hospital type (assigned using Australian hospital peer groups) [36] (Table 2). Securing ongoing funding is required for the long-term sustainability of the registry.

**Table 2:** Characteristics of Australian MT patients (n = 2451), by type of Australian peer group hospital contributing data to the MTR (n = 15) [36]

	Principal referral hospitals	Women’s hospitals	Private acute group B hospitals	Public acute group A hospitals	Public acute group C hospitals
	n = 9^a^	n = 1^a^	n = 3^a^	n = 1^a^	n = 1^a^
No. of MT cases (≥5 units in 4 h); n (%)	2158 (88.0)	15 (0.6)	46 (1.9)	206 (8.4)	26 (1.1)
No. of MT cases (≥10 units in 24 h); n (%)	911 (42.2)	8 (53.3)	9 (19.6)	83 (40.3)	6 (23.1)
Gender (male); n (%)	1378 (63.9)	0 (0)	19 (41.3)	134 (65.0)	16 (61.5)
Median age (years); [IQR]	63 [48–74]	40 [38–57]	68 [50–78]	69 [54–77]	73 [62–84]
Median hospital length of stay (days); [IQR]	18 [9–35]	9 [6–10]	15 [8–22]	12 [7–28]	6 [5–10]
Admitted to ICU; n (%)	1730 (80.2)	0 (0)	43 (93.4)	143 (69.4)	24 (92.3)
Median ICU length of stay (hrs.); [IQR]	75 [17–197]	0 (0)	75 [38–143]	48 [0–120]	85 [15–150]
Median ventilation time (hrs.); [IQR]	0 [0–52]	0 (0)	0 (0)	19 [0–134]	0 [0–5]
Survival to hospital discharge; n (%)	1706 (79.0)	15 (100)	38 (82.6)	160 (77.7)	24 (92.3)
Median RBC units in 24 h post-MT onset; [IQR]	8 [6–12]	9 [6–11]	6 [6–8]	8 [6–11]	6 [6–9]
Median FFP units in 24 h post-MT onset; [IQR]	5 [2–10]	4 [4–7]	2 [0–4]	4 [2–7]	1 [0–2]
Median Cryo units in 24 h post-MT onset; [IQR]	2 [0–10]	0 [0–6]	0 [0–6]	4 [0–8]	0 [0–0]
Median Plts units in 24 h post-MT onset; [IQR]	1 [0–2]	1[0–1]	0 [0–1]	0 [0–1]	0 [0–0]
Median RBC: FFP ratio in 24 h post-MT onset; [IQR]	1.5 [1.1–2.0]	1.5 [1.5–2.0]	2 [1.5–3.5]	1.8 [1.5–2.8]	4 [3–6]
Admission type					
Elective n (%)	649 (30.1)	6 (40.0)	44 (95.7)	50 (24.3)	5 (19.2)
Emergency n (%)	1252 (58.0)	3 (20.0)	2 (4.3)	156 (75.7)	18 (69.2)
Maternity n (%)	32 (1.5)	6 (40.0)	0 (0)	0 (0)	3 (11.5)
Unknown n (%)	225 (10.4)	0 (0)	0 (0)	0 (0)	0 (0)

^a^Numbers are counts of Australian hospitals contributing data to the MTR

### Strengths and limitations

The MTR has developed a critical mass of clinicians, laboratory scientists and other health professions regularly discussing issues around MT, including at annual investigator meetings and seminars. The MTR is an efficient and effective use of resources because it is using existing, electronically stored hospital data. It is bi-national and has representation across Australia and New Zealand from metropolitan and regional sites, including the private sector. The datasets received from sites are consistent and remove the need for an individual interpretation of patient data or selection of patients. The MTR uses an internationally recognised coding system for disease classification (ICD10 diagnosis coding) and for mapping and assigning bleeding contexts. A further strength is that the MTR offers the possibility of linkage with other registries and databases.

Although the registry has broad participation it is not yet nationally representative of Australia or New Zealand. Currently 15 Australian and 5 New Zealand sites (n = 20; 63 %) of the possible 32 sites with ethical approval are contributing data (Table 3). The three participating private hospitals were opportunistically recruited. Greater representation from the private sector is needed. One of the main limitations of the MTR is that only data existing within hospital systems can be extracted. Therefore data collection from the medical record to obtain further information, for example on medication history, adverse events or use of MT protocols, will be required for specific sub-studies.

**Table 3:** Current MTR site recruitment (n = 32)

Characteristic	No. sites n (%)
Country	
Australia	26 (81)
New Zealand	6 (19)
Australian States	
Australian Capital Territory	0
New South Wales	8 (25)
Northern Territory	0
Queensland	3 (9)
South Australia	3 (9)
Tasmania	0
Victoria	7 (22)
Western Australia	5 (16)
Public vs private	
Public	29 (91)
Private	3 (9)
Regional vs Metro	
Regional/Rural	2 (6)
Metropolitan	30 (94)
Current Australian hospital peer groups^a^	
Principal referral hospitals	16 (50)
Women’s hospitals	3 (9)
Private acute group B hospitals	3 (9)
Public acute group A hospitals	2 (6)
Combined Wwomen’s & Cchildren’s hospitals	1 (3)
Public acute group C hospitals	1 (3)
Remoteness area^a^	
Major cities	24 (75)
Inner regional	1 (3)
Outer regional	1 (3)

^a^Australian sites only (n = 26) [36]

## Conclusions

The MTR is generating data with the potential to have an impact on management and policy decision-making in CB and MT and providing benchmarking and monitoring tools for immediate application.

## Abbreviations

MTR: Massive Transfusion Registry
CB: critical bleeding
MT: massive transfusion
ICU: intensive care unit
RBC: red blood cells
FFP: fresh frozen plasma
PBM: patient blood management
NBA: National Blood Authority
NHMRC: National Health and Medical Research Council
MTP: massive transfusion protocol
TXA: tranexamic acid
HIS: hospital information services
LIS: laboratory information services
ICD10: international disease classification 10
ACHI: Australian classification of health intervention
EOC: episode of care
DRG: diagnosis-related group
NDI: national death index
CDMS: centre for data management systems
CCI: charlson comorbidity index
ACSQHC: Australian Commission on Safety and Quality in Health Care
